# Advanced Alveolar Soft Part Sarcoma Treated with Pazopanib over Three Years

**DOI:** 10.1155/2017/3738562

**Published:** 2017-10-26

**Authors:** Yoji Shido, Yukihiro Matsuyama

**Affiliations:** Department of Orthopaedic Surgery, Hamamatsu University School of Medicine, 1-20-1 Handayama, Higashi-ku, Hamamatsu, Shizuoka 431-3192, Japan

## Abstract

Alveolar soft part sarcoma (ASPS) is a rare malignant tumor that generally occurs in adolescents and young adults. It progresses slowly, but lung and brain metastases often occur in the early phase of the clinical course, and chemotherapy has been reported as not being effective for ASPS. Pazopanib is a multitargeted tyrosine kinase inhibitor that has been clinically available from November 2012 in Japan. This is a case report of a patient presented with multiple lung metastases and unresectable primary abdominal ASPS. We initially treated this patient by systemic chemotherapy with combination use of ifosfamide and doxorubicin. Stable disease was observed without any objective response. Then, we finally started to administrate pazopanib 800 mg/day. After 25 months of pazopanib administration, slight tumor reduction and a decrease of enhancement were observed. Objective responses were achieved for both the primary tumor and metastatic lung tumor; however, a newly developed brain metastasis was subsequently identified. Based on this case, pazopanib appears effective against ASPS, except for brain metastases. This case suggests that pazopanib may be useful as a first-line drug against unresectable ASPS and that longitudinal assessment of brain metastasis should be performed in similar cases.

## 1. Introduction

Alveolar soft part sarcoma (ASPS) is a rare malignant tumor that generally occurs in adolescents and young adults [1]. ASPS progresses slowly, but lung and brain metastases often occur in the early phase of the clinical course [2], and chemotherapy has been reported as not being effective for ASPS [3].

Pazopanib is a multitargeted tyrosine kinase inhibitor [4] that has been clinically available from November 2012 in Japan. Objective responses were achieved for both the primary tumor and metastatic lung tumor; however, a newly developed brain metastasis was subsequently identified. Based on this case, pazopanib appears effective against ASPS, except for brain metastases. For unresectable ASPS patients, systemic treatment using pazopanib with longitudinal assessment against brain metastasis may be feasible.

## 2. Case Report

**Figure 1:**
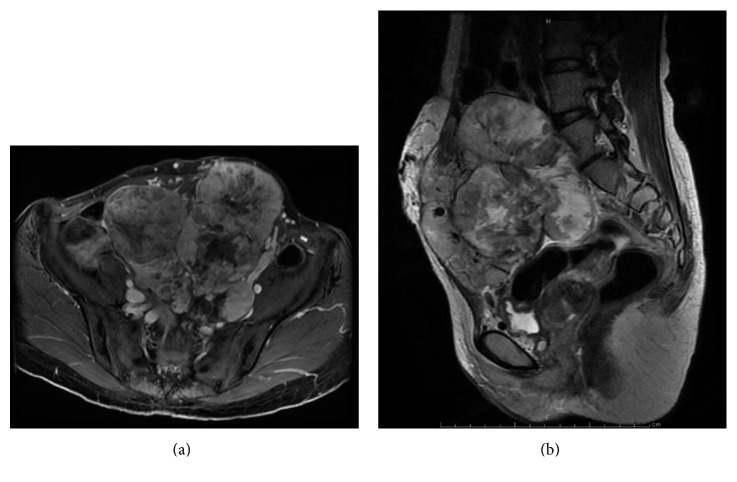
Magnetic resonance imaging findings at the initial visit. (a) T1-weighted fat suppression gadolinium-enhanced image (axial view). (b) T1-weighted fat suppression gadolinium-enhanced image (sagittal view). This MRI showed a large lobulated lesion with strong enhancement. The tumor was located at the abdominal wall, bulging to the lumbar spine.

**Figure 2:**
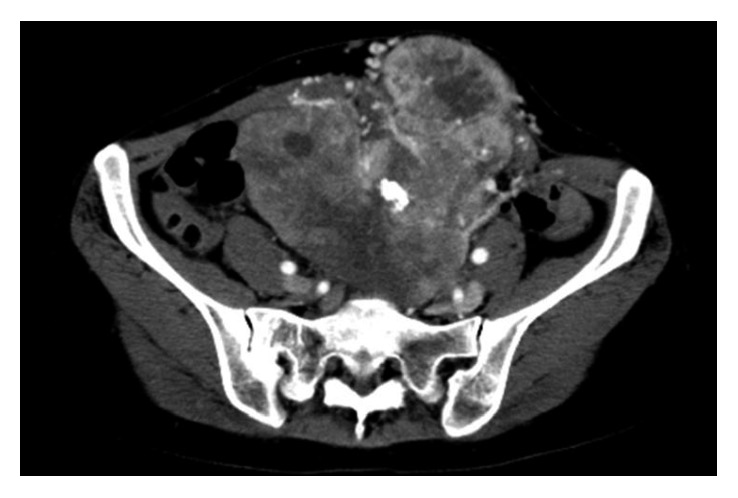
Enhanced computed tomography findings at the initial visit. CT revealed marked dot-like enhancement at the arterial phase, suggesting abundant vascular formation.

**Figure 3:**
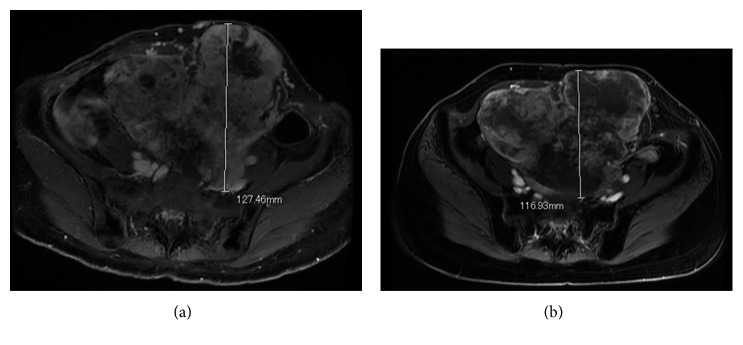
Magnetic resonance imaging findings (T1-weighted fat suppression gadolinium-enhanced image). (a) After four courses of IFM/DXR. (b) After 31 months of pazopanib administration. It was classified as no change by the RECIST criteria; however, slight tumor reduction and a decrease of enhancement were observed after 31 months of pazopanib administration.

**Figure 4:**
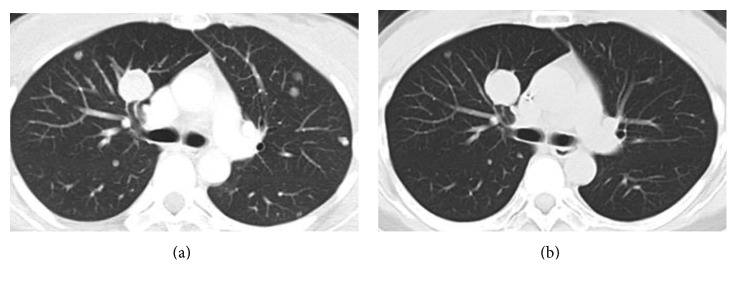
Computed tomography findings. (a) After four courses of IFM/DXR. (b) After 31 months of pazopanib administration. It was classified as no change by the RECIST criteria; however, slight tumor reduction was observed after 31 months of pazopanib administration.

**Figure 5:**
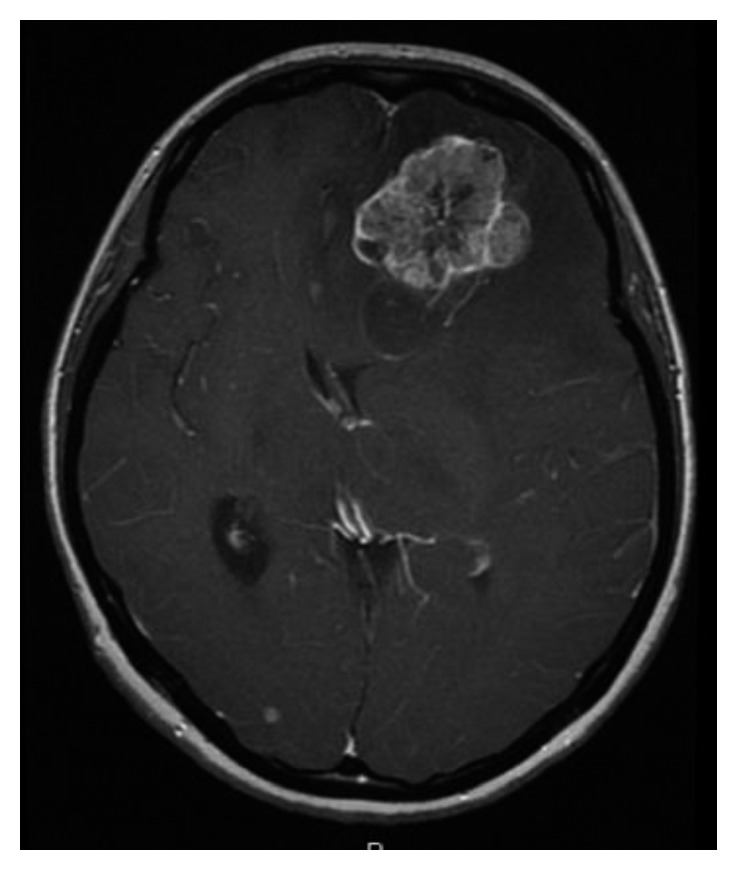
Head magnetic resonance imaging findings (T1-weighted fat suppression gadolinium-enhanced image) after 35 months of pazopanib administration. This MRI showed brain metastases with surrounding edema and a midline shift.

**Figure 6:**
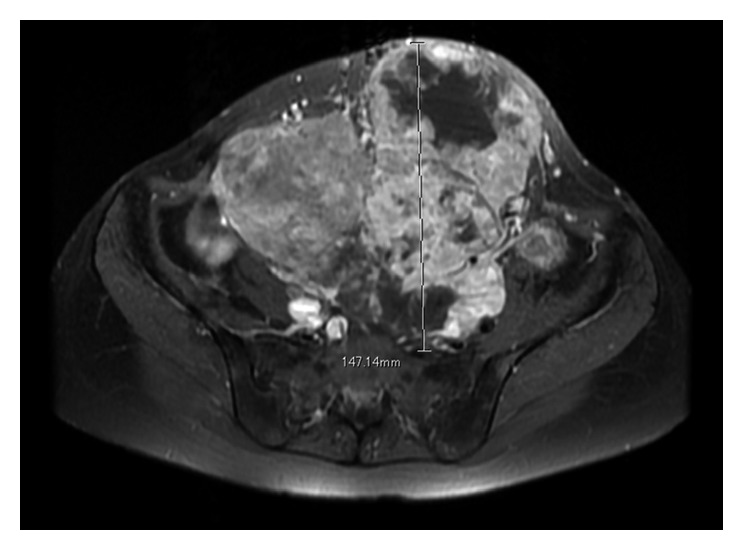
Magnetic resonance imaging findings (T1-weighted fat suppression gadolinium-enhanced image) after a drug holiday for six weeks. Tumor regrowth with increased enhancement was observed.

A 37-year-old female presented with multiple lung and abdominal masses. She had no past medical history. She had noticed an abdominal mass gradually enlarging over 10 years but had not visited a hospital. She was shown to have multiple lung masses by routine chest X-ray at a medical checkup. As a result, she visited her local hospital, where multiple lung tumors and a large abdominal tumor were found. These tumors were pathologically diagnosed as ASPS, and she was consequently referred to our hospital. At the initial visit to our hospital, a palpable soft mass in her left lower abdomen was observed. No abnormality was identified in the hematological examination, while chest X-ray showed multiple round shadows at the bilateral lung fields. Computed tomography (CT) of the chest revealed multiple lung metastases. Magnetic resonance image (MRI) showed a lobulated lesion with strong enhancement (Figure 1), and, in part of the lesion, low signal intensity was noted on T1- and T2-weighted images, suggesting flow void. The tumor was located at the abdominal wall, bulging to the lumbar spine (Figure 1). Enhanced CT revealed marked dot-like enhancement at the arterial phase, suggesting abundant vascular formation (Figure 2). Brain MRI at first presentation showed no brain metastasis. Needle biopsy was performed, and the specimen showed a nesting pattern, along with positive immunostaining of transcription factor E3 (TFE3). Moreover, fluorescence in situ hybridization examination revealed *ASPL-TFE3* gene fusion. Accordingly, the tumor was diagnosed as typical ASPS with multiple lung metastases. At this time, it was announced that pazopanib would become clinically available in the near future for second-line chemotherapy against advanced soft tissue sarcoma. Hence, we decided to initially treat this patient by systemic chemotherapy with combination use of ifosfamide (10 g/m^2^/cycle) and doxorubicin (75 mg/m^2^/cycle) (IFM/DXR) and to switch to pazopanib once it became commercially available. After four courses of chemotherapy, the response was defined as stable disease by the RECIST criteria; however, a slight enlargement of the tumor and increased enhancement effect were observed. After the four courses of combination treatment with IFM/DXR and one course of single DXR, we finally started to administrate pazopanib 800 mg/day. After 25 months of administration, a slight diminishment of the enhancement was observed. In comparison with the MRI obtained after the initial chemotherapy regimen, the response was defined as stable disease by the RECIST criteria; however, slight tumor reduction and a decrease of enhancement were observed after 31 months of pazopanib administration (Figures 3 and 3). Chest CT also showed slight reduction of the lung metastases after 34 months of pazopanib administration (Figures 4 and 4). However, after 35 months of administration, speech impairment and consciousness disturbance occurred. At this time, brain CT and MRI revealed multiple brain metastases, with surrounding edema and a midline shift (Figure 5). As a result, decompression surgery and stereotactic radiation therapy were planned, and we hence had to discontinue the administration of pazopanib to avoid incomplete wound healing. After the decompression surgery, the speech impairment was recovered. A drug holiday for six weeks was needed, after which tumor regrowth was observed (Figure 6). Similarly, regrowth of the lung metastases was also observed. Currently, the patient is taking pazopanib 800 mg/day again. At the latest follow-up, six months after restarting pazopanib, no brain bleeding and stable disease of the abdominal tumor were observed. Informed consent for publication has been obtained from the patients.

## 3. Discussion

ASPS is a rare type of sarcoma, comprising 0.5–1% of all soft tissue sarcomas, and generally occurs in teenagers and young adults aged <40 years [1]. In almost all cases, ASPS is characterized by a tumor-specific der(17)t(X;17)(p11;q25) mutation that fuses the *TFE3* gene at Xp11 to the *ASPL* gene at 17q25, creating an ASPL-TFE3 fusion protein [5]. Radical surgery is the only known cure, and standard cytotoxic chemotherapy regimens used for these soft tissue sarcomas are ineffective [2, 3, 6, 7]. Patients with localized disease at first presentation have a 71% five-year survival rate, as compared with 20% for patients with metastatic disease at the time of diagnosis [7]. In one previous study, more than half of all patients had metastasis at the time of initial clinical presentation [7], indicating a poor prognosis of ASPS patients.

ASPS is a vascular tumor, as visualized on angiography [8], and gene expression profiling studies have revealed upregulation of genes associated with angiogenesis [9, 10]. Pazopanib is a multitargeted tyrosine kinase inhibitor that targets vascular endothelial growth factor receptor, platelet-derived growth factor receptor, and c-Kit, among others [11]. The antiangiogenic activity of pazopanib through inhibition of the vascular endothelial growth factor receptor pathway might be effective against ASPS. And other tyrosine kinase inhibitors that target vascular endothelial growth factor receptors such as sunitinib and bevacizumab might be the treatment option. [12].

ASPS shows a high incidence of brain metastasis, at least 3 times higher than that of other soft tissue sarcomas [6]. In this case, a partial response was observed for the primary tumor and metastatic lung tumors, but brain metastasis occurred nevertheless. A previous animal study reported that the brain delivery of pazopanib was severely restricted [13], and it is possible that pazopanib might not pass through the blood-brain barrier in sufficient amounts. Importantly, antiangiogenic drugs such as pazopanib are supposed to be discontinued in the perioperative period to avoid wound breakage. If we had detected the brain metastasis earlier in the present case, we could have treated the patient with radiosurgery without surgical intervention. Accordingly, it is important to make a longitudinal evaluation against brain metastasis. One case report about pazopanib against ASPS was published [12], but its follow-up period was relatively short. One of the considerations for using pazopanib was high drug cost. Although it was covered by universal self-healthcare insurance in Japan, it costs over 54000 US dollars per year. In summary, to our knowledge, this is the first case report of advanced ASPS treated with pazopanib over three years. Objective responses were achieved for the primary tumor and metastatic lung tumors; however, a newly developed brain metastasis was identified. This case suggests that pazopanib may be useful as a first-line drug against unresectable ASPS and that longitudinal assessment of brain metastasis should be performed in similar cases.
